# Dorsal Root Entry Zone Lesioning for Brachial Plexus Avulsion Injuries: Case Series and Literature Review

**DOI:** 10.3389/fpain.2021.749801

**Published:** 2021-11-17

**Authors:** Alan Chalil, Qian Wang, Mohamad Abbass, Brendan G. Santyr, Keith W. MacDougall, Michael D. Staudt

**Affiliations:** ^1^Department of Clinical Neurological Sciences, London Health Sciences Centre, Western University, London, ON, Canada; ^2^Department of Neurosurgery, Oakland University William Beaumont School of Medicine, Rochester, MI, United States; ^3^Michigan Head and Spine Institute, Southfield, MI, United States

**Keywords:** brachial plexus avulsion, brachial plexus injury, deafferentation pain, DREZotomy, dorsal root entry zone lesioning, neuropathic pain

## Abstract

**Introduction:** Brachial plexus avulsion (BPA) injuries commonly occur secondary to motor vehicle collisions, usually in the young adult population. These injuries are associated with significant morbidity, and up to 90% of patients suffer from deafferentation pain. Neuromodulation procedures can be efficacious in the treatment of refractory neuropathic pain, although the treatment of pain due to BPA can be challenging. Dorsal root entry zone (DREZ) lesioning is a classical and effective neurosurgical technique which has become underutilized in treating refractory root avulsion pain.

**Methods:** A systematic review of the different technical nuances, procedural efficacy, and complication profiles regarding DREZ lesioning for BPA injuries in the literature is included. We also present an institutional case series of 7 patients with BPA injuries who underwent DREZ lesioning.

**Results:** In the literature, 692 patients were identified to have undergone DREZ lesioning for pain related to BPA. In 567 patients, the surgery was successful in reducing pain intensity by over 50% in comparison to baseline (81.9%). Complications included transient motor deficits (11%) and transient sensory deficits (11%). Other complications including permanent disability, cardiovascular complications, infections, or death were rare (<1.9%). In our case series, all but one patient achieved >50% reduction in pain intensity, with the mean pre-operative pain of 7.9 ± 0.63 (visual analog scale) reduced to 2.1 ± 0.99 at last follow-up (*p* < 0.01).

**Conclusion:** Both the literature and the current case series demonstrate excellent pain severity reduction following DREZ ablation for deafferentation pain secondary to BPA.

## Introduction

Brachial plexus avulsion (BPA) injuries are a common complication secondary to motor vehicle collisions. In one single center study, motor vehicle collisions accounted for 29% of BPA (1). The most common causative mechanism behind BPA is traction, although occasionally crushing or compression forces play a role (2). The injury is a pre-ganglionic lesion that severs axons of the nerves that form spinal nerve roots, and has been classified as one of the three major types of brachial plexus injury (3).

Dorsal root entry zone (DREZ) lesioning is an effective technique in the treatment of chronic neuropathic pain secondary to BPA that is refractory to pharmacological treatment. DREZ lesioning procedures were first described by Sindou for the treatment of pain secondary to Pancoast's syndrome (4), and multiple variations and refinements have since been made. Sindou et al. described a ventrolateral microsurgical DREZotomy approach which spares the lateral aspect of Lissauer tract (5, 6). The subsequent technique described by Nashold et al. employed radiofrequency thermocoagulation of the dorsal roots along the dorsolateral sulcus (7). The Nashold and Sindou methods report comparable efficacy of 67 and 64.7%, respectively in providing long-term pain relief (5, 7).

While the procedure was developed and popularized decades ago, it remains underutilized for the treatment of refractory pain, and the literature is limited to class III case series. In the current article, we present a review of the relevant literature, and an institutional case series of seven patients with refractory pain secondary to BPA that were treated with DREZ ablation.

## Methods

### Literature Search and Inclusion Criteria

This systematic review was conducted according to the Synthesis Without Meta-analysis guidelines. A detailed literature review was conducted through the Embase and MEDLINE databases (1947 to present) with reference scanning using the following search terms: (dorsal root entry zone OR DREZ) AND (brachial plexus) AND (injury). The references from identified articles were evaluated for the inclusion of additional studies.

Only original peer-reviewed clinical studies in humans whose results were published in the English language were considered for inclusion. Due to the relative paucity of the published literature on this procedure, we did not limit our inclusion criteria and thus included all published studies detailing the surgical management of patients diagnosed with BPA and deafferentation pain who were treated with DREZ ablation. DREZ ablation procedures included sharp dissection, laser, radiofrequency, or bipolar coagulation. Outcomes of interest included pain severity score as recorded on the visual analog scale (VAS) or as deemed appropriate in each study. Additional outcome measures included duration of pain relief, and post-operative complications.

Articles in languages other than English were excluded, as were gray literature articles. Additionally, the articles that reported the same or part of the same patient cohort at separate time points were excluded. Due to the limited literature base, the included articles were limited to case series and case reports.

### Institutional Case Series

A total of 7 patients were treated at the London Health Sciences Centre (London, Ontario, Canada) for BPA injuries (Table 1). Patients were evaluated pre-operatively using the VAS, and the following characteristics were defined before surgical intervention: pain type, distribution, and duration, baseline average, highest, and lowest pain intensity, and individualized patient targets for post-operative pain (improvement in pain intensity and activities of daily living). Statistical significance between post-operative and pre-operative VAS was calculated using a two-tailed paired *T*-test.

**Table 1:** Patient demographics for institutional case series.

**ID**	**Age**	**Sex**	**Mechanism**	**Immediate pain**	**Pain type**	**Side**	**Duration (years)**	**Pre-DREZ pain (VAS)**	**Post-DREZ pain (VAS)**	**Follow up period (Months)**	**Complications**	**Notes**
1	35	M	MVC	Yes	Pins and needles + Sharp stabbing sensation	L	1	8	0	12	None	Resolved alcohol and IVDU dependence
2	29	M	MVC	Yes	C6 allodynia + Pins and needles	R	2	5	0	12	None	Previous SCS and nerve grafting
3	30	F	MVC	Yes	Constant pins and needles + crushing	R	2	9	2	8	CSF leak and infection	Previous nerve transfer
4	33	M	MVC	Yes	Constant aching/burning + electric shock intermittently	L	5	7	0	8	Ipsilateral Trunk numbness+ knee flexion difficulty	Horner Syndrome initially
5	30	M	MVC	Yes	Constant aching/burning + electric shock intermittently	R	3	9	7	6	Ipsilateral hemibody numbness. Transient operative ataxia	Previous amputation for brachial plexus avulsion pain
6	61	M	MVC	Yes	Constant aching/burning + electric shock intermittently	L	38	7	1.5	18	Mild paresthesia in ipsilateral hemibody	Previous amputation for brachial plexus avulsion pain and nerve grafting
7	48	F	MVC	Yes	Constant crushing/burning sensation	R	2	10	4	2	Hemibody paresthesia and ataxia	Subjectively, patient reported no improvement.
Mean	38						7.6	7.9	2.1	9.4		

*IVDU, intravenous drug use; MVC, motor vehicle collision; SCS, spinal cord stimulation; VAS, visual analog scale*.

### Surgical Procedure

The patients are positioned in prone position with the head in a Mayfield clamp. A bilateral C4-T1 laminoplasty is performed using a 3 mm high speed burr and a midline durotomy is performed. The anatomy is often disrupted due to root avulsion and the formation of pseudomeningoceles at the area of interest. The spinal cord may also be rotated, depending on the severity of the avulsion injury. Once the posterolateral sulcus is identified, EMG is used to identify any motor rootlets at 1 mAmp current. A radiofrequency electrode is then used to measure impedance at the identified levels. Normal impedance is estimated at 1200–2000 Ohms, vs. 500–1000 Ohms in injured tissue. Once the injured levels have been confirmed, the probe is tilted at 30–35° from the mid- sagittal plane, and radiofrequency ablation is performed at 75°C for 15–20 s. Lesions are performed at 1–1.5 mm intervals on the side of interest, and the DREZ ablation is extended to 1–2 levels above the highest identified level (identified via visual inspection, and/or first normal impedance measurement). The dura is then closed using 6-0 Prolene, and the lamina is reattached using titanium plates and screws.

## Results

### Systematic Review

**Figure 1:**
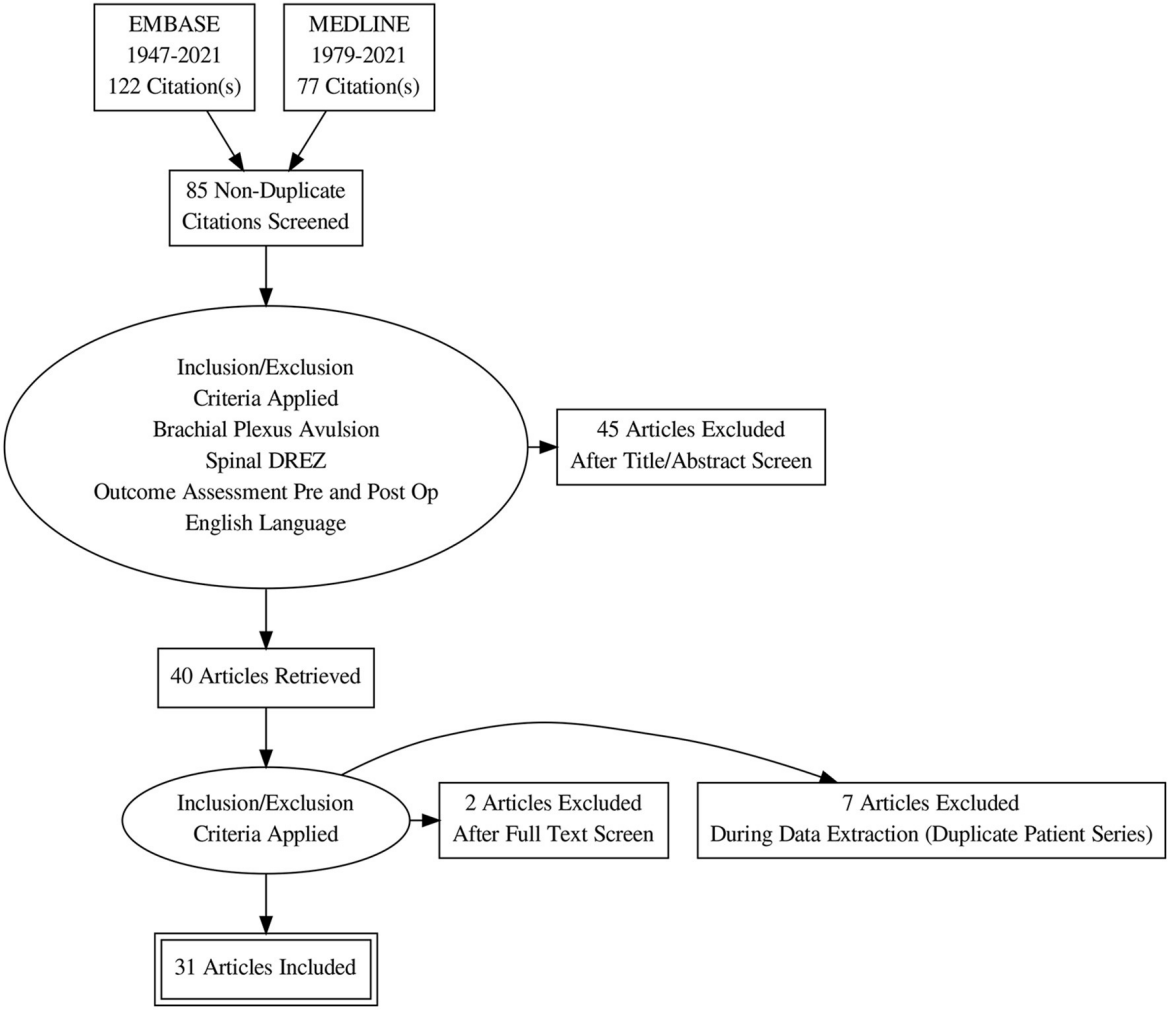
PRISMA summary of systematic review.

A total of 198 articles were identified through the literature search and underwent title and abstract review and the review process was summarized in a PRISMA chart (Figure 1). After screening by title and abstract to meet inclusion/exclusion criteria and removing duplicate publications, 85 articles underwent abstract review. All abstracts and references were examined in detail by two separate reviewers (AC and MA), and a total of 40 studies investigating brachial plexus avulsion were included in detailed analysis. Reviewer disagreement was resolved by a third reviewer (BS). Two studies were excluded (not in English language). Seven case series were based on the same patient population, and only the most recent case series published were included in further analysis (8–12).

The final study list included 31 papers, all of which were case series (6, 7, 10, 12–36) and case reports (37–39). No randomized controlled trials were identified.

The results of the literature review are summarized in Table 2. Although the VAS was used in most the studies to measure pain severity, some studies categorized outcomes as “poor,” “good,” “fair,” or “excellent.” Upon review of each study's methodology for pain intensity and outcome evaluation, all studies considered a 50% or higher improvement in post-operative pain intensity to be “good” or “excellent.” As such, study results were re-organized as “over 50% reduction in pain intensity” and “under 50% reduction in pain intensity” to reflect this pattern. For instance, if a subject's pre-operative pain was 8/10 on VAS, the subject would be considered to have achieved 50% reduction in pain intensity, if the post-operative pain was 4/10 or lower at 1 year. Missing values were excluded from the analysis. All outcomes were considered at the 1 year time point regardless of the follow up period (if longer than 1 year).

**Table 2:** Summary of articles included in the systematic review of DREZ lesioning for brachial plexus avulsion.

**Author(s), Year**	**Number of subjects**	**Pain type**	**Duration (Years)**	**Procedure**	**Pain assessment tool**	**Pre-DREZ Pain (VAS, when reported)**	**Post-DREZ Pain (VAS, when reported)**	**Pain improvement over 50% from baseline at 1 year follow-up (%)**	**Follow up period (Months)**
Nashold and Ostdahl, 1979 (7)	19	Continuous background pain, with paroxysmal electric shocks	5.9	RF	Percent improvement in pain: <25% = poor. 25–75% = fair. >75%= good	N/A	N/A	78.9%	15
Richter and Seitz, 1984 (13)	7	N/A	N/A	RF	Percentage pain improvement	N/A	N/A	71.4%	17
Bruxelle et al., 1988 (14)	24	Crushing/burning	N/A	Microsurgical DREZotomy	Percentage pain improvement	N/A	N/A	91.7%	24
Friedman et al., 1988 (16)	39	Constant burning pain or intense needle and pin sensation or crushing with paroxysmal intense pain	N/A	RF in two methods: large lesions far apart, or small lesions closer together.	Good = pain free or able to perform normal daily activities without the use of medication. Fair = pain present but required use of non-narcotic analgesics. Poor = using narcotic analgesics or if the pain limited activity	N/A	N/A	66.7%	120
Campbell et al., 1988 (15)	10	N/A	N/A	RF	Patient Interview Post op	N/A	N/A	80%	12
Ishijima et al., 1988 (17)	19	N/A	N/A	RF	Subjective percent change in pain	N/A	N/A	100%	12
Young, 1990 (18)	18	N/A	N/a	RF	Achievement of satisfactory pain relief	N/A	N/A	71.4%	12
Young, 1990 (18)	4	N/A	N/A	Laser	Achievement of satisfactory pain relief	N/A	N/A	50%	12
Jeanmonod and Sindou, 1991 (19)	3	Chronic neuropathic pain	N/A	RF	Estimated improvement from pre-operative pain	N/A	N/A	66.7%	23
Kumagai et al., 1992 (20)	6	N/A	10.8	RF	Subjective using VAS and objective via four person assessment	N/A	N/A	100%	12
Dreval, 1993 (21)	124	N/A	N/A	US	Subjective: Good, fair, unsatisfactory	N/A	N/A		47.5
Thomas and Kitchen, 1994 (10)	44	Deafferentation: constant burning or crushing nature usually affeting the whole limb in a non-dermatomal manner.	7.3	Not indicated	Follow up pain reduction assessed on a scale 0–100%, in increments of 25%	N/A	N/A	68.0%	63
Fazl et al., 1995 (22)	4	N/A	N/A	RF	Patient interview and follow up at 1, 6 and 12 months	N/A	N/A	100%	12
Rath et al., 1997 (23)	14	Constant burning in 10, constant + lancinating in 4	N/A	Thermocoagulation, 2 mm electrode, 75 degrees × 15 s, 1–2 mm apart	NA	N/A	N/A	71.4%	75.6
Samii et al., 2001 (12)	47	Constant buring crushing or electrical sensation projecting into the hand and lower arm + superimposed perceptible attack distinct from chronic pain that resulted in the need to grab the hand or arm	33.4	Cordotomy electrodes later switched to RF (75 × 15 s)	Follow up pain reduction assessed on a scale 0–100%, in increments of 25%	N/A	N/A	63.8%	168
Guenot et al., 2003 (24)	9	Continuous background pain, with paroxysmal pain crisis	6	Microsurgical DREZotomy	VAS	7.3	3.3	100%	N/A
Prestor, 2005 (26)	26	Continuous background pain, with paroxysmal electric shocks	7	C4-T1 Bipolar lesioning	VAS	N/A	N/A	96.2%	60
Sindou et al., 2005 (6)	55	Constant background pain + superimposed lancinating pain	9	Sharp incision in DREZ, 2 mm deep, angled 35 degrees medially and caudally followed by dot bipolar coagulation	Pain divided into three levels based on VAS (in person and phone interviews)	N/A	N/A	52.7%	72
Tomas and Haninec, 2005 (25)	21	N/A	N/A	RF	Percentage pain improvement. Good: 75%, fair: 25–75%, poor: 25%.	N/A	N/A	61.9%	44.1
Kanpolat et al., 2008 (27)	14	N/A	N/A	RF	VAS and Karnofsky performance scale	N/A	N/A	92.9%	12
Zheng et al., 2009 (28)	14	Thermal (burning, thrombing) or mechanical (shooting, stabbing, cramping, stinging, aching, cutting)	14.2	C5-T1 lesions using bipolar forceps	Phone interview: patients asked to assess global improvement post-surgery as a percentage	9.8	3.25	100%	15
Ali et al., 2011 (30)	11	Continuous background pain, with paroxysmal electric shocks	12.8	RF	VAS	N/A	N/A	81.8%	28
Aichaoui et al., 2011 (29)	29	Continuous background pain, with paroxysmal electric shocks	1.8	Microsurgical DREZotomy	VAS	8.8	N/A	79.3%	60
Dong et al., 2012 (31)	7	N/A	7.1	C4-T1 Bipolar lesioning	VAS	8.9	0.86	100%	12
Awad et al., 2013 (32)	10	N/A	9.6	RF DREZ	VAS	8.2	4.1	80%	78
Haninec et al., 2014 (33)	48	N/A	N/A	RF	VAS. Percent improvement >75%, 50–75%, and <50%	N/A	N/A	91.7%	24
Chivukula et al., 2015 (34)	20	Radicular, burning	2.8	Radiofrequency DREZ lesion, 75 degrees × 15 s, at 1 mm interval, depth of 2 mm	10 point numerical rating scale similar to VAS	8.1	4.1	100%	100
Son and Ha, 2015 (37)	2	Constant, crushing, stabbing, burning	N/A	RF DREZ C4-T1	VAS and personal estimate of effectiveness	8	3.5	100%	20
Ko et al., 2016 (35)	27	Constant in all, lancinating + constant in 8	7.6	RF DREZ	VAS, then categorized to: Complete, excellent = 75% or higher, good = 50–75%, poor no improvement.	N/A	N/A	81.5%	108
Piyawattanametha et al., 2017 (36)	26	Electric shock	N/A	Myelotomy with coagulation	VAS	N/A	N/A	76.9%	15
Geon et al., 2020 (38)	1	Tingling in phantom arm + electric shock sensation	27	Bipolar cautery	VAS	7	2	100%	12
Dauleac et al., 2021 (39)	1	N/A	N/A	Bipolar Cautery	N/A	N/A	N/A	100%	12

*RF, radiofrequency; VAS, visual analog scale*.

In total, 692 patients underwent DREZ ablation for brachial plexus avulsion. Of these, 567 (81.9%) patients showed a post-operative pain improvement of over 50%, while 125 patients (18.1%) had an improvement of under 50%. Only five studies reported pre- and post-operative VAS pain intensity scores: average pre-operative pain score was 8.5/10, compared to a 3.41/10 post-operative in 62 patients.

### Surgical Techniques

Radiofrequency lesioning was used in 17 studies; Samii et al. initially used cordotomy electrodes to perform the DREZ lesioning, but later reported switching to radiofrequency electrodes (12). Thermal coagulation using bipolar cautery was used in 8 studies (6, 23, 26, 28, 31, 36, 38, 39). In one study, Sindou et al. reported performing sharp incisions in the dorsolateral sulcus followed by dot coagulation using bipolar cautery (6). Laser lesioning was used in one series by Young (18), while ultrasonic lesioning was used in another series (21). No obvious differences in efficacy or complication rate among different modalities were noted. In one study, no explicit description of the procedure was provided, but previous studies by the same author indicated using radiofrequency electrode lesioning (8).

### Surgical Complications

Motor, sensory, and non-neurological complications from all studies are summarized in Table 3. Approximately 22% of all patients experienced transient or permanent neurological dysfunction (motor: 76 patients, 11%; sensory 73 patients; 10.5%). Such complications included ataxia and motor weakness in the ipsilateral lower extremities, or generalized paresthesia either in the lower extremities or hemibody. Out of all complications, 43 sensory complications were reported to be transient; however, the overall rate is difficult to calculate due to inconsistent reporting. Other reported but rare complications included wound dehiscence, infection, hemorrhage, and myocardial infarction.

**Table 3:** Literature patient outcomes following DREZ lesioning for brachial plexus avulsion.

	**Total**	**Over 50% pan relief at 1 year**	**Under 50% pain relief at 1 year**	**Motor deficits**	**Sensory deficits**	**Other**
Subjects	692	567	125	76	73	13
Percentage	100	81.9	18.1	11.0	10.5	1.9

### Institutional Case Series

Seven patients with BPA injuries underwent DREZ ablation as described above. Patient characteristics are summarized in Table 1. Five patients were male and two were female. All BPA injuries occurred secondary to motor vehicle collisions. Mean patient age was 38 years (range 29–61 years), while pain duration averaged 7.6 years (range 1–38 year). Patients were followed for an average of 9.4 months (range: 2–18 months). Four out of the seven patients received previous surgical interventions for pain management including nerve transfer, limb amputation, and/or spinal cord stimulation.

Most patients reported similar pain characteristics including a consistent baseline crushing/burning pain with intermittent electrical pain. Mean pre-operative pain intensity ± standard error on VAS was 7.9 ± 0.63. All but one patient achieved >50% reduction in pain intensity, with an average post-operative pain of 2.1 ± 0.99 on the VAS at the last follow up [t_(6)_ = 7.83, *p* < 0.01]. One patient subjectively reported little to no improvement, although their pain intensity score on VAS was reduced by 60% following surgery.

Regarding post-operative complications, several patients reported hemibody paresthesias and transient ataxia, but only one found these sensations to be disabling and required long term rehabilitation. One patient suffered a cerebrospinal fluid leak and her post-operative course was later complicated by wound dehiscence and infection requiring operative revision; however, the patient remained satisfied with her pain improvement.

## Discussion

BPA is a devastating injury caused by over-stretching the brachial plexus, which results in pulling the C5-T1 nerve rootlets from their origin in the spinal cord. Almost 90% of patients with BPA suffer from post-traumatic deafferentation pain (40). Such injuries cause significant motor and sensory deficits, and are a major cause of morbidity in affected patients.

The case series presented here, although limited to seven patients, demonstrates the efficacy of the DREZ ablation procedure in treating neuropathic pain secondary to BPA injuries. All but one patient (85.7%) showed a >50% reduction in pain intensity following DREZ ablation. This aligns with previously published studies, which demonstrate 81.9% of patients achieving overall good post-operative pain control. Pre-operative pain in our series ranged between 7 and 10/10 on the VAS, which closely resembles literature values. This provides an objective measurement of the severity of deafferentation pain and its impact on patients' quality of life.

### Relevant Anatomy and Pathophysiology

The DREZ aligns with the posterolateral sulcus on the spinal cord and is composed of Lissauer's tract and the dorsal horn (5). Lissauer's tract is composed of axons of nociceptive fibers that run in the rostro-caudal plane along the dorsal horn, giving off branches into the horn at each level. This tract modulates signal transduction through nociceptive fibers, which plays an important role in pain perception. Nociceptive fibers travel through the dorsal root to the dorsal horn and terminate in Rexed laminae 1, 2, and 5—neurons within these laminae modulate sensory and pain signals.

Several theories have been proposed to describe the pathophysiology of chronic pain associated with BPA. As BPA is a preganglionic injury, it affects the function of second-order neurons in the dorsal horn cells, which forms the basis of the deafferentation process. In the event of deafferentation and avulsion injuries, the interneurons located in the dorsal horn exhibit epileptiform-like activity (41, 42). This hyperactivity may occur in the dorsal horn neurons at the level of the avulsed rootlets, or even at adjacent spinal levels (24, 43). Sindou proposed lesioning the lateral aspect of the DREZ in patients with cancer-related pain as a means to interrupt interneuron hyperactivity as well as longitudinal pain signal conduction. Consequently, Sindou's proposed surgery was extended to treat BPA, phantom limb pain, hemi-body pain, and post-herpetic neuralgia, to varying degrees of efficacy (5).

MRI is considered the best non-invasive diagnostic modality for BPA, but its reliability has been controversial (2). As a result, the gold standard to differentiate between pre-ganglionic and post-ganglionic brachial plexus injury is surgical exploration (44). Generally, pre-ganglionic BPA is treated primarily with nerve transfer or grafting, usually using the intercostal nerve (3).

As the affected population is generally young and healthy, long-term disability and disease burden becomes significant from an individual and societal level. Notably, up to 90% of patients with true root avulsion suffer from deafferentation pain, and 20% of these patients eventually will require surgical intervention due to intractable pain (amputation, nerve transfer, spinal cord stimulation, or other surgery) (7, 29). Deafferentation pain is characteristically described as constant and crushing, accompanied with episodic sharp pain that radiates distally down the arm (45). The degree of deafferentation pain has been described to be correlated with the number of avulsed nerve roots (45). Early and successful surgical repair of BPA has been shown to provide relief from intractable deafferentation pain (46).

Indeed, prior to developing refractory deafferentation pain, many patients have undergone nerve grafting, suturing, and neurotization in attempt to delay or prevent such pain onset; however, despite early surgical intervention, pain is the primary symptom that has the greatest negative impact on quality-of-life (44). Neuropathic pain medications are almost universally initiated, although deafferentation pain tends to be refractory. As a result, surgical management is often necessary for symptomatic relief. Neuromodulation procedures such as spinal cord stimulation have been described as efficacious (47–49); however, DREZ ablation is generally considered superior in the treatment of BPA-related deafferentation pain.

### The Efficacy of DREZ Ablation

The pain response to DREZ ablation may be multifactorial. Our literature review demonstrated good overall efficacy of DREZ specifically for BPA injuries. One case series pointed to lower efficacy of DREZ in BPA patients who received surgical amputations in comparison to those who experienced traumatic amputations (28). In the same series, only 40% of patients with phantom limb pain and intact nerve roots responded well to DREZ lesions, while all patients with phantom limb pain and root avulsion responded well.

Several factors were hypothesized to be associated with better outcomes following DREZ ablation, but no consistent trends were highlighted in our literature review. Some evidence suggests that patients with longer duration of deafferentation pain (6 years or more) may experience better pain relief following DREZ ablation compared to patients with shorter duration (3 years or less) (6, 26). Both Piyawattanametha et al. and Zheng et al. found that spinal root avulsion and the number of avulsed roots is associated with better pain response from DREZ ablation (28, 36). This fits well with the pathophysiology of deafferentation pain which forms the theoretical basis of DREZ ablation; however, it is important to stress that DREZ ablation is a destructive procedure based on interruption of normal nociceptive pathways. This raises the risk of pain recurrence, or the formation of new neuropathic pain in the years following surgery.

Interestingly, Samii et al. failed to find a significant correlation between pre-operative pain duration or number of avulsed roots and the response to DREZ ablation (12). It may be that patients with a higher number of avulsed roots require a correspondingly higher number of intra-operative DREZ lesions, which in turn produce a more efficacious or sustained post-operative pain response. The dermatomal distribution of deafferentation pain usually corresponds to the avulsed brachial plexus nerve roots (16, 26); however, this hypothesis cannot be verified based on our literature review.

Our case series and literature review confirm both the efficacy and relative safety of DREZ ablation for BPA. When combining cases from the literature review, nearly 11% of patients described transient or permanent paresthesia in the ipsilateral lower extremity, likely due to thermal injury to the dorsal columns during surgery. A further 11% of patients experienced transient or permanent motor deficits in the ipsilateral lower extremity, almost all in the form of ataxia. This effect may result from injury to the spinocerebellar tract, or may be secondary to loss of proprioception in the leg that was misclassified as a motor deficit. Other possible complications that may not be specific to DREZ ablation surgery include cardiac complications, surgical wound infection, CSF leak, or postoperative hemorrhage.

## Limitations

The most significant limitation of this review lies in the quality of research studies examining the efficacy of DREZ for chronic pain in general, including brachial plexus avulsion injuries. No randomized controlled trials were identified in the literature, and the majority of the studies included in this review are observational and retrospective in nature. Furthermore, while all studies measured pre-operative and post-operative pain intensity, only six studies (including our case series) provided objective VAS scores. Most studies expressed results in percentage change. This significantly limits the ability to compile data from different studies to extrapolate more robust conclusions. Additionally, there is a large variation in the length of follow-up among reported studies, which limits our ability to fully ascertain the long term effects of the procedure. Regardless, this review provides the most comprehensive and up-to-date evidence regarding the efficacy of DREZ ablation surgery for management of BPA.

## Conclusion

BPA is a devastating injury with lasting neurological sequelae. Deafferentation pain adds to the morbidity of these injuries and tends to be refractory to common neuropathic analgesia regimens. This case series and detailed literature review reinforces that DREZ ablation offers an effective and safe treatment of deafferentation pain secondary to BPA at 1 year post-operatively, despite its underutilization. The current literature lacks consistency in outcome measures, follow-up periods, and use of operative techniques. More structured and detailed studies, in the form of prospective cohort studies, are needed in the future.

## Data Availability Statement

The original contributions presented in the study are included in the article/supplementary material, further inquiries can be directed to the corresponding author.

## Ethics Statement

Ethical review and approval was not required for the study on human participants in accordance with the local legislation and institutional requirements. Written informed consent for participation was not required for this study in accordance with the national legislation and the institutional requirements.

## Author Contributions

AC and MS contributed to conception and design of the study. AC, MA, and BS performed the literature review. MA performed the statistical analysis. AC and QW wrote the first draft of the manuscript. MS and KM provided supervision and project administration. All authors read and approved the submitted version.

## Conflict of Interest

The authors declare that the research was conducted in the absence of any commercial or financial relationships that could be construed as a potential conflict of interest.

## Publisher's Note

All claims expressed in this article are solely those of the authors and do not necessarily represent those of their affiliated organizations, or those of the publisher, the editors and the reviewers. Any product that may be evaluated in this article, or claim that may be made by its manufacturer, is not guaranteed or endorsed by the publisher.
